# Potentiating Biosynthesis of Alkaloids and Polyphenolic Substances in *Catharanthus roseus* Plant Using ĸ-Carrageenan

**DOI:** 10.3390/molecules28083642

**Published:** 2023-04-21

**Authors:** Hossam S. El-Beltagi, Salwa M. El-Sayed, Ahmed. N. Abdelhamid, Karim. M. Hassan, Walaa. A. Elshalakany, Mona Ibrahim Nossier, Nadiyah M. Alabdallah, Nadi Awad Al-Harbi, Salem Mesfir Al-Qahtani, Doaa Bahaa Eldin Darwish, Zahid Khorshid Abbas, Hemmat A. Ibrahim

**Affiliations:** 1 Agricultural Biotechnology Department, College of Agriculture and Food Sciences, King Faisal University, Al-Ahsa 31982, Saudi Arabia; 2 Biochemistry Department, Faculty of Agriculture, Cairo University, Giza 12613, Egypt; 3 Department of Biochemistry, Faculty of Agriculture, Ain Shams University, Cairo 11566, Egypt; 4 Department of Horticulture, Faculty of Agriculture, Ain Shams University, Cairo 11566, Egypt; 5 Soil and Water Department, Faculty of Agriculture 11241, Ain Shams University, Cairo 11566, Egypt; 6 Department of Biology, College of Science, Imam Abdulrahman Bin Faisal University, P.O. Box 1982, Dammam 31441, Saudi Arabia; 7 Basic & Applied Scientific Research Centre, Imam Abdulrahman Bin Faisal University, P.O. Box 1982, Dammam 31441, Saudi Arabia; 8 Biology Department, University College of Tayma, University of Tabuk, Tabuk 47512, Saudi Arabia; 9 Biology department, Faculty of Science, University of Tabuk, Tabuk 71491, Saudi Arabia; 10 Botany Department, Faculty of Science, Mansoura University, Mansoura 35511, Egypt

**Keywords:** *Catharanthus roseus*, ĸ-carrageenan, polyphenolic substances, flavonoids, vincristine, Vincamine, Catharanthine, HPLC

## Abstract

*Catharanthus roseus* is a medicinal plant that produces indole alkaloids, which are utilized in anticancer therapy. Vinblastine and vincristine, two commercially important antineoplastic alkaloids, are mostly found in the leaves of *Catharanthus roseus*. ĸ-carrageenan has been proven as plant growth promoting substance for a number of medicinal and agricultural plants. Considering the importance of ĸ-carrageenan as a promoter of plant growth and phytochemical constituents, especially alkaloids production in *Catharanthus roseus*, an experiment was carried out to explore the effect of ĸ-carrageenan on the plant growth, phytochemicals content, pigments content, and production of antitumor alkaloids in *Catharanthus roseus* after planting. Foliar application of ĸ-carrageenan (at 0, 400, 600 and 800 ppm) significantly improved the performance of *Catharanthus roseus*. Phytochemical analysis involved determining the amount of total phenolics (TP), flavonoids (F), free amino acids (FAA), alkaloids (TAC) and pigments contents by spectrophotometer, minerals by ICP, amino acids, phenolic compounds and alkaloids (Vincamine, Catharanthine, Vincracine (Vincristine), and vinblastine) analysis uses HPLC. The results indicated that all examined ĸ-carrageenan treatments led to a significant (*p* ≤ 0.05) increase in growth parameters compared to the untreated plants. Phytochemical examination indicates that the spray of ĸ-carrageenan at 800 mg L^−1^ increased the yield of alkaloids (Vincamine, Catharanthine and Vincracine (Vincristine)) by 41.85 μg/g DW, total phenolic compounds by 3948.6 μg gallic/g FW, the content of flavonoids 951.3 μg quercetin /g FW and carotenoids content 32.97 mg/g FW as compared to the control. An amount of 400 ppm ĸ-carrageenan treatment gave the best contents of FAA, Chl a, Chl b and anthocyanin. The element content of K, Ca, Cu, Zn and Se increased by treatments. Amino acids constituents and phenolics compounds contents were altered by ĸ-carrageenan.

## 1. Introduction

Medicinal plants have a long history of usage in traditional medicine. Ethno-botanical information on medicinal plants and their usage by indigenous cultures is useful in the conservation of traditional cultures, biodiversity, community health care and drug development. *Vinca rosea* (*Catharanthus roseus*) is an important medicinal plant belonging to the Apocynaceae family; this plant is a dicotyledonous angiosperm and synthesizes two indole alkaloids: vinblastine and vincristine that are used to fight cancer [1,2]. Plant phenolic substances protect against a variety of environmental stresses, including excessive sun radiation, disease and insect attack [3]. Furthermore, these phytochemicals may act as strong antioxidant agents, assisting in the defense against ageing and health risks in humans [4].

Carrageenans are linear sulfated polysaccharides, extracted from several marine red algae [5]. These compounds are a linear chain of d-galactose residues linked by α-(1, 3) and β-(1, 4) glycosidic bonds which are substituted with ester sulphonic groups per two repeating units of galactose. Carrageenans is divided into three classes according to the presence of sulphate group per disaccharide units, where koppa (κ)-carrageenan contains one sulphate group, iota (ι)-carrageenan has two sulphate groups and lambda (λ)-carrageenan has three sulphate groups [6,7]. ĸ-carrageenan is composed mainly of 1-linked β-d-galactopyranosyl-4-sulfate residues alternating with 4-linked 3, 6-anhydro-α-d-galactopyranosyl residues forming the repeating disaccharide unit of its structure, while disaccharide units in i-carrageenan consist of β-d-galactopyranosyl-4-sulfate residues linked to 3, 6-anhydrogalactopyranosyl-2-sulfate residues. On the other hand, the repeated disaccharide unit in λ– carrageenan consists of 1-linked β-d-galactopyranosy l-2-sulfate with 4 –linked β-d-galactopyranosyl-2, 6-disulfate residues [8,9].

Carrageenans enhance plant growth by regulating various metabolic processes involved in photosynthesis, cell division, and purine and pyrimidine biosynthesis and also involved in assimilation of sulfur and nitrogen. Carrageenans also induce plant defense responses against biotic stress by modifying the various pathways of defense activities, such as signaling pathways of ethylene, jasmonate and salicylate [10]. *ĸ*- and i-carrageenans treatments on Eucalyptus globulus caused increase in plant height by 58 and 47% and increase in trunk diameter by 44 and 40% over the control [11]. Additionally, Bi et al. [12] found that ĸ-carrageenan encourages growth improving and early flowering in chickpea and maize where it increases leaves number in both plants, but it increases plant height and the number of pods in chickpea only. ĸ-carrageenan promoted defense response in plants, induced signaling and defense gene expression in plants. These effects may be due to its high content of sulphate groups [10].

Thus, the aim of this study was to investigate whether foliar application with ĸ-carrageenan affects growth parameters and phytochemical constituents in *Catharanthus roseus* plants.

## 2. Results

### 2.1. Effect of Carrageenan Spraying on Growth Parameters of Catharanthus Roseus

**Figure 1:**
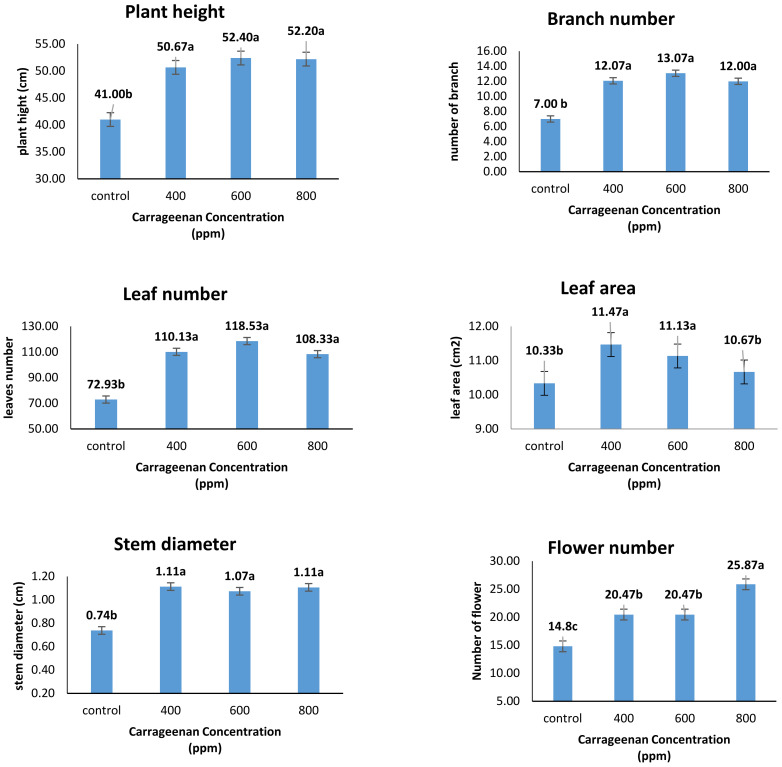
Effect of Carrageenan spraying on vegetative growth of *Catharanthus roseus*. Plant height (cm), lateral branches number, leaves number, leaf area (cm^2^), stem diameter (cm) and number of flowers. Different letters indicate significant differences according to Duncan’s multiple range tests (*p* < 0.05).

From the results obtained, it was found that there was a clear increase in both plant height, number of branches and leaves, leaf area, stem circumference and number of flowers when using different concentrations of the extract (400, 600 and 800 ppm) compared to the control (Figure 1). It was also noted that concentration 400 ppm gave the best in the effect on leaf area (11.47 cm^2^) compared to the rest of the concentrations used, while the flowers number was elevated by 800 ppm carrageenan treatment (25.87). There were no significant differences between carrageenan treatments (400, 600 and 800 ppm) in plant height, branch number, leaves number and stem diameter.

### 2.2. Effect of Carragennan Spraying on Chemical Parameters of Catharanthus roseus

Carrageenan treatments increased all phytochemical constituents. The *Catharanthus roseus* content of total phenolic compounds (TPC), flavonoids (F) and alkaloids (TAC) increased directly by increasing the concentration of ĸ-carrageenan from 400 to 800 ppm (Table 1), where 800 ppm treatment gave the highest values (3948.6 μg/g FW, 754.6 μg/g FW and 3518 μg/g DW), respectively, in comparison with control, which gave the lowest values (2476.5 μg/g FW, 271.7 μg/g FW and 1359 μg/g DW), respectively. An amount of 400 ppm carrageenan spraying treatment only gave a significant increase in free amino acids content (195.57 mg/g FW), with no significant differences between other treatments.

### 2.3. Effect of Carrageenan Treatments on Pigments

**Figure 2:**
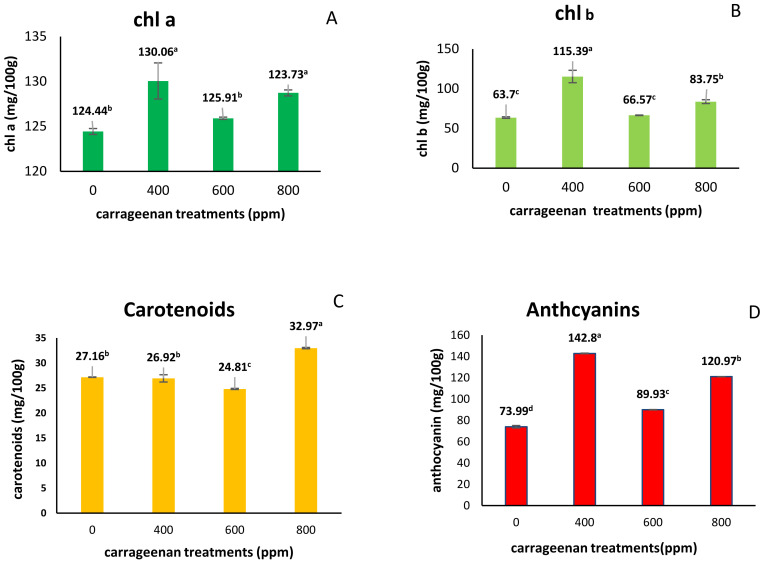
Effect of Carrageenan spraying on pigments (chl a, chl b, carotenoids and anthocyanins) contents in Catharanthus roseus. (**A**) chl a; (**B**) chl b; (**C**) carotenoids; (**D**) anthocyanins. Different letters (small lettes) indicate significant differences according to Duncan’s multiple range tests (*p* < 0.05).

When studying the effect of carrageenan on pigments (Figure 2), data illustrated that vinca plants treated with ĸ-carrageenan 400 record the highest contents of chl a, chl b and anthocyanins (130.06 mg/100 g FW,115.39 mg/100 g FW and 142.8 mg/100 g FW) in comparison with control, followed by 800 ppm treatments, while carotenoids content score the highest significant increase in plants treated with 800 ppm ĸ-carrageenan (32.97 mg/100 g FW), in comparison with control (27.16 mg/100 g FW).

### 2.4. Effect of Carrageenan Treatments on Elements Content

From the results presented in Table 2, we found a clear effect on the concentration of some of the macro, micro and essential elements inside the plant when using different concentrations of carrageenan.

#### 2.4.1. Macro Elements (N, P, K and Ca)

There was a significant increase in the concentration of nitrogen (N) inside the plant after the use of ĸ-carrageenan at a concentration of 800 ppm (16.42), but spraying with concentrations of 400–600 ppm caused a significant drop in the concentration of N (13.36 and 14.66), respectively, compared to the control (15.58), while vinca content of phosphorous (P) was not affected by all ĸ-carrageenan treatments. On the other hand, there was significant and clear elevation in potassium (K) and calcium (Ca) concentrations when using ĸ-carrageenan with its different concentrations, especially the 600 ppm concentration (1.38 and 1.65) compared to the control (0.29 and 0.25), respectively (see Table 2).

#### 2.4.2. Micro and Essential Elements (Mn, Cu, Zn and Se)

In addition, the micro elements and essential elements of Catharanthus roseus were investigated (Table 2). When using the concentration of ĸ-carrageenan 400 ppm, it caused a significant decrease in manganese (Mn) content (46.14), but ĸ-carrageenan 600 and 800 ppm spraying elevated manganese concentration compared to the control (76.98), especially spraying with a concentration of 600 ppm (154.56). Copper (Cu), zinc (Zn) and selenium (Se) concentrations were elevated by all ĸ-carrageenan treatments compared to the control (7.48, 15 and 32.43), respectively. ĸ-carrageenan treatment of 400 ppm had the highest values of Cu, Zn and Se (78.79, 54.22 and 117.17).

### 2.5. HPLC Analysis of Free Amino Acids, Phenolic Compounds and Alkaloids of Catharanthus roseus Leaves Treated with k-Carrageenan

HPLC analysis of free amino acids (Table 3) illustrated that 17 amino acids were identified in all treatments, which are as follows: aspartic (ASP), glutamic (GLU), serine, histidine, glycine, therionine, arginine, alanine, tyrosine, valine, methionine, tryptophan, phenylalanine, isoleucine, leucine, lysine and proline. Cysteine was not found in all treatments. Proline was the highest amino acids in all treatments, while histidine was the lowest amino acid in all treatments. Plants at control treatments had the higher content of ASP (1.55), GLU (4.01), serine (5.41), arginine (9.21), alanine (6.85), tyrosine (3.14) and lysine (1.42) in comparison with ĸ-carrageenan treatments. ĸ-carrageenan treatments increase *C. roseus* content of histidine, glycine, threonine, valine, methionine, tryptophan, phenylalanine, isoleucine, leucine and proline, whereas 800 ppm treatment gave the highest content of histidine (0.26), glycine (1.85), threonine (6.82), valine (4.93), methionine (5.00), tryptophan (2.44), phenylalanine (4.28) and proline (65.28), while plants treated with 400 ppm ĸ-carrageenan had a higher content of isoleucine (3.36) and leucine (6.32).

Phenolic compounds (PC) analysis by HPLC (Table 4) showed that 16 phenolic compounds were identified in *C. roseus* leaves, which are as follows: gallic acid, chlorogenic acid, methyl gallate, caffeic acid, syringic acid, rutin, ellagic acid, coumaric acid, vanillin, ferulic, naringenin, daidzein, quercetin, cinnamic acid and apigenin, while pyrocatechol, kaempferol and hesperetin contents were absent in all treatments. Chlorogenic was the highest compound in all treatments, followed by methylgallate, syrinigic and querectin, while coumaric, ferulic, naringenin and cinnamic had the lowest values in all treatments.

At the results, caffeic scored the highest level in control treatment (0.64), while apigenin was found in control treatment only with low concentration (0.03). Methylgallate and rutin were elevated by 400 ppm ĸ-carrageenan treatment only (5.16 and 1.11, respectively) while their contents at 600–800 ppm treatments were lowered than control. Ellagic acid was found only in 400 and 600 ppm ĸ-carrageenan treatments (0.89 and 0.3, respectively). The 600 and 800 ppm ĸ-carrageenan treatments increased coumaric and cinnamic, where 800 ppm treatments gave the highest levels (0.22 and 0.24, respectively). All ĸ-carrageenan treatments led to an increase in the content of the rest of phenolic compounds, as 800 ppm treatment contained the highest concentrations of chlorogenic (18.97), ferulic (0.74), naringenin (0.3), daidzein (1.88) querectin (3.73) and cinnamic (0.24), while vanillin recorded the highest level in both 400 and 800 ppm ĸ-carrageenan treatments (0.22 and 0.23) without any significant difference.

Carrageenan treatments had an effect on biosynthesis of HBA derivatives such as gallic, methylgallate, syringic and ellagic acid (Table 4), where they caused a decrease in gallic acid level while they caused an increase in syringic acid content. Additionally, ellagic acid appeared in 400 and 600 ppm treatments only, while 400 ppm treatment caused an increase in methylgallate content. These results may be due to all carrageenan treatments inducing syringic acid synthesis from gallic acid, while 400–800 ppm carrageenan treatments encourage ellagic acid synthesis from gallic acids. Additionally, part of gallic is converted to methylgallate in plants treated with 400 ppm only. Hydroxycinnamic derivatives (HCA) were influenced by carrageenan treatments, where all carrageenan treatments cause increase in ferulic and chlorogenic content. While cinnamic and coumaric levels were increased by 600–800 ppm treatments, only caffeic was decreased by carrageenan treatments. These results may be due to that carrageenan encourages conversion of phenylalanine to cinnamic acids (CA) and hydroxycinnamic derivatives (HCA), where phenylalanine converts to cinnamic acid with phenylalanine ammonialyase through the phenylpropanoid pathway, then CA converts HCA derivatives via hydroxylation and methylation processes where *p*-coumaric is formed from CA by hydroxylation, followed by caffeic by another hydroxylation process and then ferulic acid by methylation process. Carrageenan may be enhancing all enzymatic processes so synthesis of cinnamic, *p*-coumaric and ferulic were increased. The reason for a decrease in level of caffeic may be due to its conversion to chlorogenic acid, whose level was increased by carrageenan treatments.

**Figure 3:**
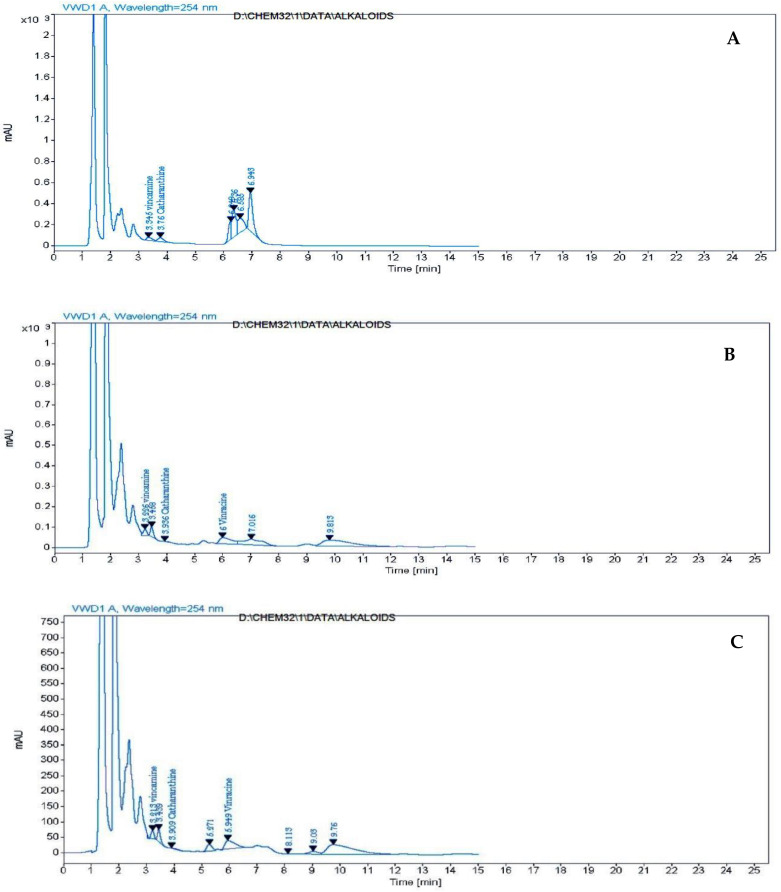

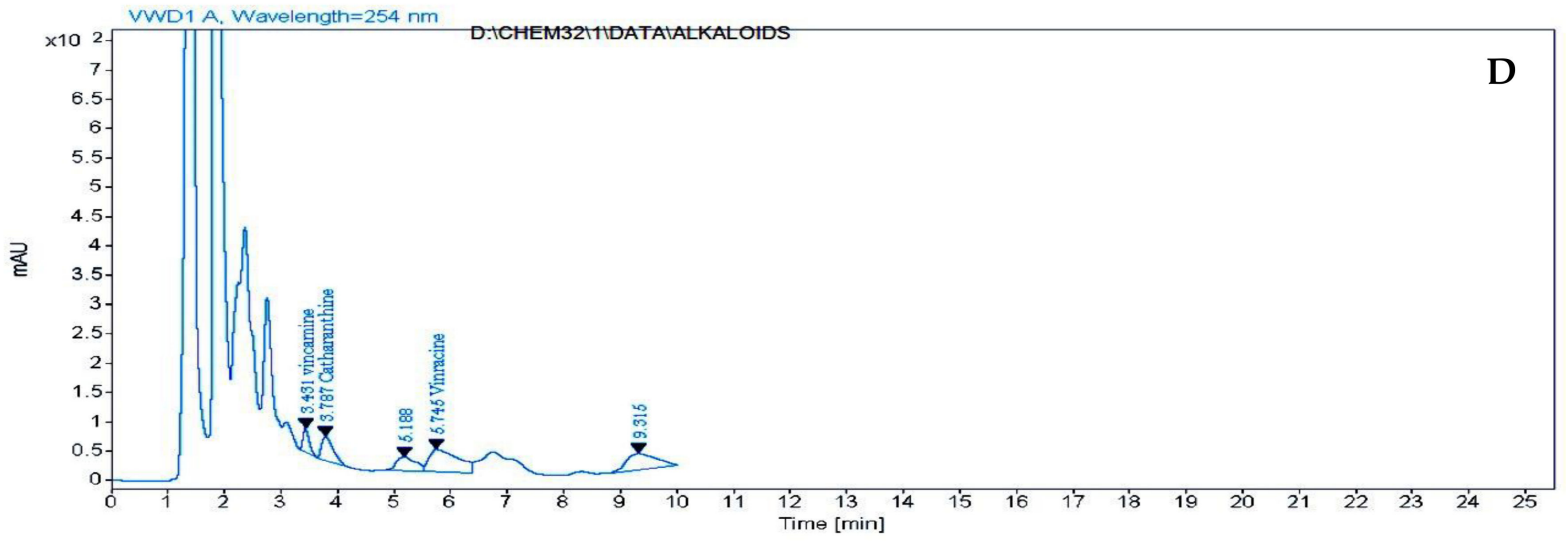
HPLC chromatograms of alkaloids (**A**) control (**B**) T1; *Catharanthus roseus* treated with 400 ppm carrageenan (**C**) T2; *C. roseus* treated with 600 ppm carrageenan (**D**) T3; *C. roseus* treated with 800 ppm carrageenan.

Data in Table 5, Figure 3 and Supplementary Materials illustrated that untreated plants contained the highest content of vincamine (24.911 μg/g), respectively, followed by plants treated with 800 ppm, 600 ppm and 400 ppm treatment, which contained the lowest contents of these compounds (12.043 μg/g). While catharanthine scored the highest value of a plant sprayed with 800 ppm of carrageenan in comparison with other treatments, vincracine (vincristine) appeared in carrageenan treatments only, where it scored the highest content at 800 ppm treated plants (13.522 μg/g). Vinblastine was absent from all treatments. In general, 800 ppm treatment contains the highest total alkaloid content as a sum of three alkaloids (vincamine, catharanthine and vincristine).

## 3. Discussion

*Catharanthus roseus* were cultured on different concentrations of carrageenan, and the effect of alkaloids accumulation, phytochemical constituents and growth parameters were studied.

### 3.1. Effect of Carrageenan Spraying on Growth Parameters of Catharanthus roseus

Our study showed an increase in plant height, number of branches and leaves, leaf area, stem circumference and number of flowers when using different concentrations. These results were in harmony with the investigation of Naeem et al. [13], who found that the spray of gamma-ray irradiated carrageenan (at 0–100 ppm) elevated the *Catharanthus roseus* productivity and significantly improved the performance of *C. roseus.* an amount of 80 ppm of irradiated carrageenan caused an increase of 35.4% in leaves yield and 37.4% herbage yield. The increase in leaf area by carrageenan treatment increased sunlight harvesting, CO_2_ consumption and chlorophyll content, which in turn increased the rate of photosynthesis which led to dry matter accumulation in plants [13]. Additionally, Mousavi et al. [14] concluded that 1 g L^−1^ carrageenan treatments elevated basil shoot length and leaf area 1 g L^−1^ carrageenan treatments stimulates growth of basil by increasing shoot length and leaf area and an increase in phenylalanine amonnialyase activity, phenolic compounds, lignin levels and antioxidant activity in basil. Therefore, it can said that carrageenan led to activiation of the phenylpropanoid pathway. The reason for this can be attributed to the fact that carrageenan is rich in sulfur, which is a kind of linear sulfated polysaccharide [15].

Sulfur plays an important role in plant metabolism, as it is necessary in the synthesis of plant proteins, amino acids and some vitamins and enzymes. This was confirmed by Stewart [16] that is is used in the formation of amino acids, proteins and oils. It is necessary for chlorophyll formation, promotes nodulation in legumes and helps develop and activate certain enzymes and vitamins, which lead to an increase in plant growth and yield. Sulfur is one of the water-soluble elements, and therefore it is in the extract of carrageenan that is soluble and therefore easily absorbed by the leaves of the plant [17]. Sulfur (S) is important in plant development, where it necessary for many metabolities biosynthesis such as amino acids containing sulfur, glutathione, thiamine, coenzyme A, flavonoids and phenolic compounds and phytochelatins [18,19].

Sulphur is found the proteinaceous amino acids such as methionine and cysteine, glutathione, chlorophyll, coenzyme A and S-adenosyl-methionine [20,21]. There is also a close relationship between sulfur and nitrogen, as the presence of sulfur increases the efficiency of plant absorption. The relationship between S and N is not surprising since both are components of protein and are involved in chlorophyll formation. They are also linked by the role of S in the conversion of nitrate to amino acids. Crops having high N need will usually also have high S needs [16]. Nitrogen increases the rate of photosynthesis, which results in an increase in cell divisions and the synthesis of proteins and amino acids that encourage an increase in plant heights and an increase in the number of branches, leaves, leaf area and stem circumference. Nitrogen is very important and needed for plant growth. In addition, it encourages the uptake and utilization of other nutrients including potassium and phosphorous and controls overall growth of the plant [22,23].

### 3.2. Effect of Carragennan Spraying on Chemical Parameters and Phytochemical Screening of Catharanthus roseus

In our study, *Catharanthus roseus* plants which were treated with carrageenan had an increase in all phytochemical constituents such as total phenolic compounds (TPC), flavonoids (F) and alkaloids (TAC), which increased directly by increasing the concentration of ĸ-carrageenan. Similarly, results were matching with the finding of Ahmad and Tahir [24], who illustrated that TPC was increased in *Iris germanica*, *Iris kashmiriana* and *Iris ensata* during the flowering stage. The highest contents of TPC and F were observed in the flowers and leaves of heather at the flowering stage [25].

Our results showed that 400 ppm carrageenan spraying treatment only gave a significant increase in free amino acids content, with no significant differences between other treatments. These results are due to carrageenan rich with sulfur, which plays an important role in plant metabolism, as it is necessary in the synthesis of plant proteins, amino acids and some vitamins and enzymes. It is also necessary for chlorophyll formation [16] and consequently leads to an increase in amino acids content.

Amino acids have many important roles in plants as they increase the cell capability to absorb water and nutrients from soil and in this way, they increase the vegetative growth; in addition to the increase in proteins anabolism, they contribute to multiple functions of plant metabolism and improve carbon assimilation rate which results in enhancement of total dry matter and consequently increase production [26,27]. Plant amino acids improve protein synthesis, cell division, plant pigments, indole acetic acid, gibberelli and ethylene contents [28,29]. These increase amino acids content, especially phenylalanine, which turned to phenolic compounds and flavonoids through phenylpropanoids pathway and phenylpropanoids acetate pathway, respectively [25], which causes an increase in the content of phenolic compounds and flavonoids. Phenolic compounds play an important role in the regulation of flower development and antioxidant defense mechanism by scavenging free radicals and preventing the flower from oxidative stress [30]. On the other hand, the increase in amino acids content, especially tryptophan, led to an increase in alkaloids synthesis, which results in an increase in the content of alkaloids [13].

### 3.3. Effect of Carrageenan Treatments on Pigment

Pigment content increased in *Catharanthus roseus* plants treated with ĸ-carrageenan 400 ppm in comparison with control, followed by 800 ppm treatments. These results agreed with the finding of Naeem et al. [13] who said that 80 ppm of gamma-ray-irradiated carrageenan treatment increased chlorophyll and carotenoids content by 16.6% and 7.18% in *C. roseus* leaves. ĸ-carrageenan treatments increased the leaf area (Figure 2), which resulted in an increase in sunlight harvesting, CO_2_ consumption and chlorophyll content, which in turn increased the rate of photosynthesis which led to dry matter accumulation in plants [13]. These results are due to carrageenan rich with sulfur which increases nitrogen (N) absorption. N is considered a part of the chlorophyll molecule, which gives plants their green color and is involved in creating food for the plant through photosynthesis, which leads to increased plant growth and yield [30]. Sulfur presence in carrageenan plays an important role in the synthesis of amino acids [16]. S and Se were correlated with N absorption and assimilation. N assimilation is associated with synthesis of amino acids, proteins, phytohormones, phenylpropanoids, alkaloids, etc. [31,32].

These increases in amino acids synthesis, especially phenylalanine, which turned to phenolic compounds. Phenolic compounds are converted to anthocyanin through the phenylpropanoid–acetate pathway. Anthocyanin gives a purple color to vinca flowers. The highest anthocyanin content was detected in the flower, which is important in pollinating insects’ attraction, therefore increasing plant reproduction [25]. Anthocyanins are natural pigments which belong to flavonoids that are responsible for the color of flowers. They have a wide range of colors such as purple, blue, orange and red. In addition, they protect the plant from harmful UV rays [33,34]. Carrageenan treatments cause an increase in Se level (Table 2), which causes accumulation of carotenoids. For example, *Lycium chinense* leaves seedling treated with selenite (20 mg L^−1^) contain high level of carotenoids in comparison with control [35]. This increase in carotenoids is due to the fact that Se led to an increase in genes expression of enzymes responsible for carotenoids biosynthesis [36].

### 3.4. Effect of Carrageenan Treatments on Elements Content

We found a clear effect on the concentration of some of the macro, micro and essential elements inside the plant when using different concentrations of carrageenan. The reason for this can be attributed to the fact that carrageenan is rich in sulfate that interacts with many of elements. Sulfur is known to interact with almost all essential macronutrients, secondary nutrients and micronutrients. These interactions can either enhance or reduce growth and yield of crops by influencing the nutrient uptake and utilization. There is a close relationship between sulfur and nitrogen, as the presence of sulfur increases the efficiency of plant absorption. The relationship between S and N is not surprising since both are components of protein and are involved in chlorophyll formation. They are also linked by the role of S in the conversion of nitrate to amino acids. Crops having high N need will usually also have high S needs [16].

Sulfur and phosphorus are both essential elements and are taken up by plants in the anionic form from the soil. The requirement of plants for these elements is similar [37]. The role of potassium and sulfur in augmenting the yield and improving the quality of crops is well known [38,39,40]. Increasing absorption of K leads to an increase in flowers number in plants. Zinc is an important micronutrient, which enters the plant primarily via absorption of Zn^+2^ by roots from soil solution. Interaction of sulfur with zinc has been extensively investigated on seed and dry matter yield of many crops [41]. The relationship between S and Se is well known. In fact, as the Se content of the fertilizer increases, the S uptake and concentration in the plant decrease. Sulphate in the growth solution reduced selenate uptake by plants and increased the S content of the leaves. Under low sulphate treatment there was a clear correlation between leaf S content and shoot Se content across the genotypes, thus indicating that the overall activity of the S transport systems also determines Se transport. The difference in Se content between the low and the high sulphate treatments was significantly higher in shoot than in root, confirming that the Se translocation from root to shoot is probably more affected by high sulphate supply than Se uptake by root. Consequently, spraying vinka with carrageenan (400 ppm), which results in an increase in the sulfur content of the shoot, leads to an increase in the concentration of Se in the shoot. While concentration of carrageenan increased from 600–800 ppm caused a decrease in Se, it is still higher than control because the antagonistic effect of S with Se. Sulfur treatment causes an increase in some microelements uptake (such as Mn, Cu and Zn) which is caused by a higher availability of these elements due to the acidifying effect of elemental sulfur [42,43].

### 3.5. HPLC Analysis of Free Amino Acids, Phenolic Compounds and Alkaloids of C. roseus Leaves Treated with ĸ-Carrageenan

Amino acids are a constructive unit for enzymes and structural proteins synthesis and are precursors of secondary metabolites containg nitrogen such as alkaloids and act as precursors of phenolic compounds, flavonoids and anthocyanin [44]. The results were in harmony with Park et al. [45], who observed increasing amino acids content during flower development of *Lycoris radiata*, including glutamine, asparagine, glutamic acid, aspartic acid, threonine, valine, tyrosine, isoleucine, glycine, cysteine, serine and beta-alanine. Lysine, phenylalanine, tryptophan, methionine and leucine levels were elevated in the fully opened flower stage. Additionally, Borghi and Fernie [46] reported that some amino acids such as proline, phenylalanine, tyrosine, tryptophan, neutral, basic and acidic amino acids were found in nectar and petal peptide, while proline neutral, basic and acidic amino acids were in sepals and pollen grains. Arginine was concentrated in mitochondria of pollen grains and petals.

In the results, in the aspartic family, there was a decrease in aspartic and lysine, while methionine and threonine increased by ĸ-carrageenan treatments (Table 3). These results may be due to an increase in aspartate conversion to asparagine, methionine, threonine and isoleucine, while decreasing conversion to lysine. Asparagine is an storage compounds of N that is used in energy generation during ovule maturation and embryo growth [47]. During pollen grain development, asparagine was hydrolyzed by asparaginases A1 and B1 to ammonium, which was assimilated to give glutamine, which was amino acids biosynthesis [48,49]. Threonine increases plant immunity in the flowering stage, so it increases by carrageenan treatments. On the other hand, lysine contributes in the plant stress response to abiotic and biotic stress [50,51], so it decreased in *C. roseus* by carrageenan treatments because *C. roseus* plants are not exposed to stress. In addition, lysine catabolism products enter the TCA cycle to provide plants with energy [52]. Isoleucine is a branched amino acid that acts as an osmotic regulating agent [53], and it is considered a precursor in b-alanine synthesis in plants [54]. Some amino acids contained sulfur such as cystine, cysteine and methionine which are similar in containing sulfur in the side chain of their structure [55]. Some amino acids contain sulfur involved in plant proteins and maintaining the infrastructure or formation of the active sites of enzymes [51]. Sulfur contributes in disulfide bonds formation (-S-S-) between polypeptide chains, which is important in determining protein shape and structure. Methionine contributes to ethylene formation and has an effect on root growth [46].

In the serine family, there was an increase in glycine content, while serine decreased from ĸ-carrageenan treatments. Cysteine disappeared in all treatments (0, 400, 600 and 800 ppm). This result may be increasing conversion of serine to glycine. Glycine is a major building block of chlorophyll within the plants. It helps to increase the chlorophyll concentration, resulting in higher metabolism [56]. Additionally, glycine cause increasing photosynthesis efficiency, chlorophyll anabolism and plant growth. In addition, glycine has a role in pollination [44]. This is consistent with the results obtained in chlorophyll content (Figure 2) and growth parameters (Figure 1). Cysteine disappeared in all treatments because of its involvement in plant proteins and maintaining the infrastructure or formation of the active sites of enzymes [56]. Serine increase chl biosynthesis and regulates plant hormone balance [46].

In the glutamic family, glutamic and arginine levels descended while histidine and proline content ascended in plants treated with ĸ-carrageenan (Table 3). These results may be due to high glutamic conversion to histidine and proline, while its conversion to arginine is decreased. Proline synthesis also occurs in flowers, where proline is found in pollen grains and nectar and also in the protein composition of pollen coats [57]. Free proline protects pollen from drying out [58], while proline in nectar may supply pollinating insects with energy [59]. Proline regulates osmotic potential, maintains the protoplasm colloidal properties and eliminates the harmful effects of free radicals [46]. Histidine causes an increase in shoot growth and early production [46]. Histidine biosynthesis associates with nucleotide metabolism across 5′-phosphoribosyl-1-pyrophosphate, which is an intermediate metabolite of anthranilate [60]. Anthranilic acid is an important precursor of tryptophan and IAA synthesis [61]. Tryptophan in *C. roseus* is important in the biosynthesis of indole alkaloids. The arginine decrease is due to its contribution to chlorophyll biosynthesis, root production, cell division, and polyamide anabolism [62].

In the alanine family, the level of alanine decreased while valine and leucine increased in plants treated with ĸ-carrageenan (Table 3). ĸ-carrageenan may increase conversion of pyruvate to valine and leucine, where branched amino acids (isoleucine, valine and leucine) are important in plant growth, the stress resistance, and flavor compound biosynthesis in plants [63]. Valine has effects on plant growth, roots and seed production [46]. β-alanine consumed in pantothenate synthesis is then converted into coenzyme A. Coenzyme A is involved in the metabolism of lipids and carbohydrates [64].

In the aromatic amino acids family, both phenylalanine and treptophan increased, while the level of tyrosine decreased from ĸ-carrageenan treatments. This means that ĸ-carrageenan causes an increase in the synthesis of tryptophan and phenylalanine. Aromatic amino acids phenylalanine, tyrosine, and tryptophan play many important roles in plants where they are considered essential components of protein synthesis and convert to many growth hormones and secondary metabolites [65]. Tyrosine may be consumed as a precursor of many metabolites which had varied physiological roles such as non-protein amino acids, attractants and defense compounds [66]. Tryptophan is an essential component in the synthesis of a large number of biologically active compounds, such as terpenoid indole alkaloids and auxin, which is essential for plant growth and early productivity [65,67,68].

Phenolic compounds (PC) are secondary metabolites, which contain one or more phenol ring with at least one hydroxyl group [69]. PC biosynthesis begins from L-phenylalanine through the shikimic acid and phenylpropanoid pathways [70]. They are found in most plant organs, play an important role in plant resistance to biotic and abiotic stresses [70,71], and are considered strong antioxidants [72], so they consequently play important roles in many human diseases, including anti-cancer, anti-inflammatory, anti-diabetic, anti-allergic, anti-Alzheimer’s and they protect the nervous system, liver and cardiovascular system [73]. Our results were in harmony with the investigation of Park et al. [45], who found that chlorogenic acid had the highest phenolic compounds content (222.52 μg/g DW) in *Lycoris radiata* followed by caffeic (104 μg/g DW), 4-hydroxybenzoic acid (51.82 μg/g DW), gallic acid (34.63 μg/g DW), respectively, while flowers had the lowest content of *p*-coumaric, sinapic acid and ferulic acid (1.1, 0.05 and 0.02 μg/g DW) in the fully opened flower stage.

Foliar application with carrageenan provides plants with sulphur, which is an important element for biosynthesis of antioxidants, cofactors, secondary metabolites, amino acids (cysteine and methionine) [74] and S-adenosylmethionine, which is a cofactor [75,76]. SAM plays important role in transferring the CH_3_- group to various molecules for synthesis of many secondary metabolites such as alkaloids, phytosterols, osmoprotectants, precursors of lignins, suberins, hydroxycinnamic acids, flavonoids, anthocyanins and stilbens [19].

ĸ-carrageenan spraying increased Cu and Zn levels in *C. roseus* (Table 2), which caused an increase in phenolic and flavonoids contents. Osmane Badiaa et al. [77] concluded that different treatments of Cu and Zn (200–500 ppm) on tomato leaves and roots caused proline accumulation and induced antioxidants synthesis such as polyphenols and flavonoids. Additionally, carrageenan treatments increased Se concentration in *C. roseus* (Table 2). This increase in Se caused an increase in phenol and flavonoid contents [78]. Selenate spray treatment (10 μM Se) resulted in an increase in hydroxycinnamic acids in basil leaves by 1.6 times in comparison to untreated plants [79]. This increase in phenolic compounds content is due to that Se increases phenylalanine ammonia-lyase activity, which is a main enzyme in the phenyl propanoids pathway [80]. The increase in amino acids, especially phenylalanine (Table 3), which is converted to phenolic compounds through phenylpropanoid pathway, leads to an increase in phenolic acids, flavonoids and anthocyanins. Additionally, the increase in the protein content, which probably includes an increase in the enzymatic proteins, means an increase in the synthesis of enzymes that participate in the synthesis of phenolic compounds. On the other hand, an increase in Se content may affect the level of gene expression of enzymes involved in the synthesis of phenolic compounds, flavonoids and anthocyanin [81]. Phenolic acids are of two groups, hydroxyl benzoic acids (HBA) and hydroxycinnamic acids (HCA). HBA are synthesized from 3-hydroxyshikimate or from chorismate pathway and can synthesized from cinnamic acid via phenylpropanoids pathway. Additionally, HCA can be synthesized from the phenylpropanoids pathway [82,83].

Ellagic acid is a more effective antioxidant, due to its four hydroxyl groups and lactone present in its structure [84], which are necessary for scavenging hydroxyl and super oxide anion radicals [85] and in binding to DNA and DNA protection from alkylating injury [86]. Gallic and ellagic are important in biosynthesis of gallotannins and ellagitannins, respectively, which protect plant from insect injury. Gallic and methyl gallate have strong antifungal activity [87]. Syringic acid has a free radical scavenging activity due to two methoxy groups at positions C3 and C5 in the aromatic ring [88]. In addition, it contributes to lignin structure [89]. Chlorogenic acid is an ester of caffeic acid and quinic acid and an intermediate in lignin biosynthesis; it is considered an anti-insect and herbivore defence compound in plants [90,91]. Carrageenan treatments also increase conversion of ferulic to vanillin by vanillin synthase (Table 4), which increases from treatments. Vanillin is an aldehyde of vanillic acid and contains aldhyde, hydroxyl group and methoxy group on C1, C4 and C3, respectively, at the aromatic ring. Vanillin is considered an active inducer of *Rhizobium* node genes, and it had antimicrobial activity. Additionally, vanillin had high efficacy on nodule number, nodule mass and acetylene reduction activity in plant [92].

Flavonoids have a three-ring structure in the C6–C3–C6 form, divided into many groups such as flavanones (naringenin and hespertin), flavanols (catechin), flavonols (guerectin and kaempferol), flavones (apigenin), isoflavones (diadzein) and anthocyanidins [88]. Flavonoids have important roles in plants, such as scavenging reactive oxygen species (ROS) to maintain the redox state in plant tissues, giving colors to flowers and other parts, and contributing in the auxin transport process [93,94]. Flavonoids protect plants against pathogen attacks and herbivores. The antioxidant properties are due to presence of conjugated double bonds and functional groups in the rings [95]. Flavonoids are present in a bilayer of lipid in cell walls and regulate both enzymatic and non-enzymatic lipids peroxidation [96].

Flavonoids biosynthesis altered by carrageenan treatments, where they cause an increase in querectin and daidzein, while the naringenin level was increased by 600–800 ppm treatments only. On the other hand, rutin was elevated by 400 ppm treatments only, while apigenin was absent from carrageenan treatments. Catechin, kaempferol and hesperetin were absent from all treatments in addition to control (Table 4). These results may be due to that carrageenan encourages conversion of *p*-coumaric acid to naringenin through the phenylpropanoids–acetate pathway. Naringenin is the initiator of the synthesis of the rest of flavonoids. It plays many important roles in plants, such as protecting the plant from UV-B radiation [97], attracting insects for pollination, and contributing to the defense system [98]. It is clear from the results that the treatments with carrageenan led to an increase in the synthesis of flavonoids molecules containing two adjacent hydroxyl groups on the B ring such as querectin and rutin, which have a higher antioxidant activity than flavonoids compounds containing one hydroxyl group on the B ring such as apigenin and kaempferol, which disappear from carrageenan treatments. Rutin is a quercetin glycoside and plays an important role in plants’ protection against UV radiation or pathogens [99]. This means that during the flowering stage, many free radicals are formed, so plants resort to the synthesis of flavonoids that contain dihydroxy substituting groups in the B ring, which have higher antioxidant activity than flavonoids that contain one hydroxyl group in the B ring. Flavonoids which have dihydroxy substituting groups in the B ring had higher antioxidant activity but lower UV absorption capacity than their monohydroxyl group in the B ring [93,100].

Although the catechin contains two hydroxyl groups on the B rings, it disappeared in all vinca. This is due to catechin converting to epigallocatechin or epigallocatechin gallate, which are necessary for signal processing in seed development and inducing plant resistance to disease by promoting jasmonic acid signal [101,102,103,104,105,106] or may be catechin converts to glycoside form. Catechin derivatives are considered anti-insect and herbivore defence compound in plant [107]. Daidzein is isoflavone, which is different than apigenin (which belongs to flavone) in the position of the B ring, which is in the three position in isoflavone while it is in the two position in flavone. Diadzein plays an important role in the interaction between the plant and the environment, and it is considered as a phytoalaxin [108,109].

Plant alkaloids are secondary metabolites, characterized by the presence of a nitrogen atom in its composition, other than the amide and peptide bonds. Natural precursors of alkaloids are synthesized from various metabolites, mainly amino acids. The function of alkaloids in plant is related to the formation of seeds and defense of plant from pests [110]. Vinblastine and vincristine are the major alkaloids accumulated in vinca plants. They belong to the class of bisindole alkaloids and are synthesised from tryptophan, which loses amino groups and converts them to tryptamine. Then, tryptamine reacts with secologanin to form catharanthine through several steps. After formation of catharanthine, it is transformed into vindoline and/or vincamine in several steps. The concentration of these compounds varies according to the gene expression of the enzymes responsible for the synthesis of either of them. Then, catharanthine reacts with vindoline by anhydrovinblastine synthase and produces α-3′,4′-anhydrovinblastine, which is converted into vinblastine and then further converted into vincristine (vinracine). The enzymes contribute in the formation of vinblastine and vincristine are not known [111,112,113]. However, spraying with 400 ppm gave the lowest content of catharanthine and it resulted in decrease in the synthesis of vincamine, which resulted in an elevation in vincristine biosynthesis. The catharanthine biosynthesis began to gradually increase again by 600 ppm carrageenan treatment followed by 800 ppm and accordingly, the created amount of vincramine and vincristine increased from the previous treatment. These results due to 800 ppm carrageenan treatments caused an increase in free amino acids biosynthesis, especially tryptophan (Table 3), which in turn led to an increase in indole alkaloid biosynthesis [13] via increasing the enzymes activity, which catalyzes conversion of catharanthine to vincristine. Additionally, treatment with carrageenan caused an increase in the level of Se (Table 2), which in turn led to an increase in alkaloids content [114,115]. Carrageenans cause modification to various pathways of defense activities, such as signaling pathways of ethylene, jasmonate and salicylate [10]. This led to an increase in ethylene production. Ethylene caused accumulation to catharanthine and vindoline, which are precursors to vinblastine and vincristine synthesis in *C. roseus* [116]. Jasmonates are plant-signaling molecules which induce the synthesis of indole alkaloid pathway in the hairy roots of *C. roseus* [117].

*Catharanthus roseus* alkaloids such as vincristine, vinblastine, vindesine, vinorelbine, vinflunine and vindesine) consider microtubule-targeting agents which prevent continuous mitotic divisions and cancer cells growth [118]. Vincristine is the alkaloid most widely used in pediatric cancer treatment programs and has been incorporated into many acute chemotherapy regimens for the treatment of leukemia and tumors of the nervous central system [119]. Vincristine also causes peripheral nurotoxicity in pediatric tumors [120].

## 4. Material and Methods

### 4.1. Plant Material, Field Experiment and Treatments

One month old, healthy and uniform in shape vinca (*Catharanthus roseus* L.) transplants (20 cm, length) were purchased from a private nursery, Giza, Egypt. The experimental layout was of complete randomized design (CRD) with 3 replicates. All the experimental pots were distributed as follows: 4 Carrageenan treatments × 5 pots × 3 replicate = 60 plants. Each single transplant was cultivated in the first week of March (2021) in a plastic pot (35 cm diameter) filled with peat moss and sand (1:1). The irrigation was regularly executed 2–3 times a week after calculating the decrease in water-holding capacity using the weight method. Fertilization was also executed using a half-strength Hoagland’s nutrient solution (one time every 10 days). After 2 months of cultivation, all pots (60 pots) were divided into 4 groups in the first week of May to apply the foliar applications of ĸ-carrageenan (Carrageenan; Sigma Aldrich, St. Louis, MO, USA) at 0 (distilled water as a control), 400, 600, and 800 ppm. Each group of plants (15 pots) was sprayed 5 times with 15 mL of a specific concentration of ĸ-carrageenan solutions. In the first week of July, plants were gathered to determine the growth parameters and phytochemical constituents.

### 4.2. Preparation of 80% Ethanolic Extract

**Figure 4:**
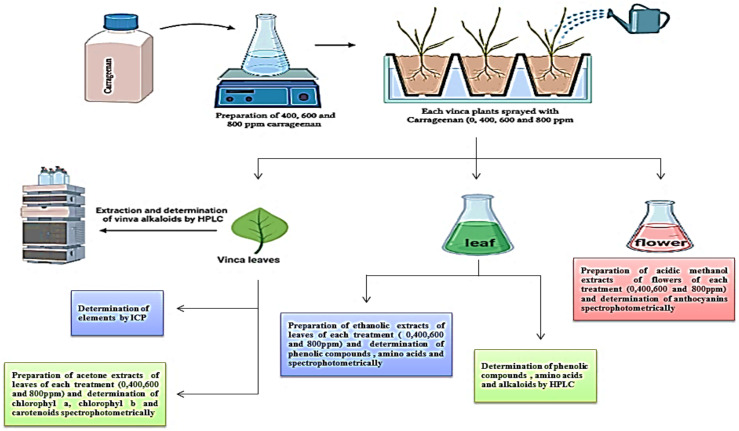
Experimental design and phytochemical constituents screening.

The lyophilized samples of leaves, flowers and roots of *Catharanthus roseus* (5 g) were extracted with 50 mL of ethanol 80% for 24 h at 4 °C, followed by the extracts being centrifuged for 5 min at 6000 rpm. The residue was re-extracted in the same manner three times and the three supernatants were combined and evaporated under vacuum at 40 °C in a rotary evaporator; the remaining extracts were lyophilized to be exploited in phytochemical qualitative screening (Figure 4).

### 4.3. Phytochemical Screening of the Catharanthus roseus Leaf Extracts

Ethanolic extracts of leaves of *Catharanthus roseus* were subjected to quantitative analysis of some phytochemicals in *Vinca* such as total phenols content (TP); flavonoids content (F); free amino acids content (FAA); total alkaloids content; Chl a, Chl b and carotenoids contents; anthocyanin content and vincristine and vinblastine (Figure 1).

#### 4.3.1. Quantitative Analysis of Some Phytochemicals in the *Catharanthus roseus* Leaf

0.5 g of fresh leaves was macerated in 10 mL 80% ethanol for at least 24 h at 5 °C, the alcohol was collected and the remaining tissue re-extracted with 10 mL 80% ethanol about three times. At the end, the collected extract was completed to 50 mL using 80% ethanol. This extract was prepared for phenolic compounds, flavonoids and free amino acids determination (Figure 1).

##### Total Phenols Content (TP)

The total phenols content were measured according to the method referred to in Folin–Ciocalteu [120]. An amount of 1 mL of ethanolic extract 80% was mixed and thoroughly shaken in a test tube with 0.5 mL of Folin–Ciocalteu phenol reagent. After 3 min, 1 mL of Na_2_CO_3_ (20%) was added to the mixture, and 10 mL of distilled water was added to the amount. The reaction was let to proceed for 1 h. A blank solution was prepared in place of the sample with 1 mL of distilled water. The absorbance at 725 nm was measured after 1 h using (UV-Vis spectrophotometer UV 9100 B, LabTech, LabTech, Inc. 114 South Street, Hopkinton, Massachuse 01748 USA). Gallic acid was used as the standard solution. Total phenolic compound was calculated as mg/100 gFW.

##### Flavonoids Content (F)

Flavonoids content was determined by the AlCl_3_ colorimetric method as described by Marinova et al. [121]. An amount of 1 mL of ethanolic extract 80% was transferred to a 10 mL test tube, which included 4 mL of distilled water. Then, 0.3 mL NaNO_2_ (5%) was added. An amount of 0.3 mL of AlCl_3_ (10%) was added after 5 min. At the 6th min, 2 mL NaOH (1M) was added, total volume was made up to 10 mL with distilled water, and the absorbance was quantified against blank at 510 nm. The concentration of total flavonoids was calculated as mg/100 g FW using the standard curve of Quercetin.

##### Free Amino Acids Content (FAA)

Free amino acids content was determined colourimetrically by using ninhydrin solution according to Jayaraman [122] using lysine as a standard. The amino acids were calculated as mg/100 g FW as follows: take 1 mL of ethanolic extract 80% and 1 mL ninhydrin (2 g dissolved in 25 mL acetone and 25 mL acetate buffer 0.2 M, pH 5.5) in screw test tube which includes 3 mL of distilled water. Cover test tubes and place them in boiling water bath for 15 min. Cool through tap water, then add 5 mL of ethanol 50%. The produced blue purple color was measured at 570 nm against blank.

#### 4.3.2. Total Alkaloids Content

Total alkaloids content was measured by the method as mentioned by Shamsa et al. [123]. The plant tissues (5 g) were ground and extracted with methanol for 24 h in a continuous extraction apparatus at 26 °C. The extract was filtered and methanol was evaporated by rotary evaporator under vacuum at 45 °C to dry. Part of this residue was dissolved in HCl 2 N and then filtered. One mL of this solution was transferred to a separate funnel and washed with 10 mL chloroform (3 times). The pH of this solution was adjusted to be neutral with NaOH 0.1 N. Then, 5 mL of bromocresol green (BCG) solution and 5 mL phosphate buffer were added to this solution. The mixture was shaken and the complex formed was extracted with 1, 2, 3, 4 mL chloroform by vigorous shaking. The extracts were collected in a 10 mL volumetric flask and diluted to volume with chloroform. The absorbance of the complex in chloroform was measured at 470 nm. The concentration of total alkaloids was calculated using the standard curve of atropine.

#### 4.3.3. Chl a, Chl b and Carotenoids Contents

Chlorophyll-a (Chl a), Chlorophyll-b(Chl b) and carotenoids were determined according to Sumanta et al. [124]. An amount of 50 mL of acetone (80%) was used to homogenise 0.5 g of fresh leaf in a tissue homogenizer, then filtered using Whatman filter paper No. 1. an amount of 0.5 mL of supernatant is mixed with 4.5 mL of acetone (80%). Then, absorbance at three wavelengths 663.2, 645.8 and 470 nm were measured against blank (acetone 80% only) at a spectrophotometer. The concentration of Chlorophyll-a (Chl a), Chlorophyll-b (Chl b) and carotenoids (Cx + c) were calculated from the following equations.

Chl a = 12.25A663.2 − 279A646.8(1)

Chl b = 21.5A646.8 − 5.1A663.2(2)

Cx + c = (1000A470 − 1.82Chl a − 85.02Chl b)/198(3)

#### 4.3.4. Anthocyanin Content

An amount of 0.1 g fresh flowers was crushed in 10 mL acidified methanol [methanol: HCl 1N (95:5, *v*/*v*)]. Then, it was soaked for 24 h at room temperature, and a centrifugation was executed at 4000× *g* for 10 min, and the supernatant was taken and adjusted to 10 mL with the same extraction solution. Anthocyanin content was measured according to Giusti and Wrolstad [125]. Then, was used two buffer solutions system of different pH: KCl buffer pH 1.0 (25 mM) and sodium acetate buffer pH 4.5 (0.4 M). In brief, 1 mL of the extract was combined with 4 mL of each of the two buffers separately. The absorbance was recorded at 510 and 700 nm after incubation for 15 min at room temperature. Anthocyanin content was calculated as mg cyanidin-3-glucoside equivalent/100 g FW by the following equation.

Anthocyanin content = A × MW × DF × 1000/Ɛ × Molar(4)

where A: Absorbance = [(A510 nm–A700 nm)] pH1.0—[(A510 nm–A700 nm)] pH 4.5; MW: Molecular weight (449.2 g/mol); DF: Dilution Factor; Ɛ: Molar extinction coefficient of cyanidin-3-glucoside (26,900 L Mol^−1^·cm^−1^).

#### 4.3.5. Elements Contents

The plant samples were oven dried at 70 °C, and then wet digested by a mixture of H_2_SO_4_ and H_2_O_2_ according to the method described by Cottenie et al. [126]. Total N content was determined by the micro Kjeldahl method using 5% boric acid and 40% NaOH according to A.O.A.C. [127]. Total K, P, Se, Mn, Zn and Cu were determined using ICP Mass Spectrometry [128].

### 4.4. Free Amino Acids Separation by HPLC

A 2 g sample was macerated in 10 mL methanol 70% for 24 h at 50 °C. After cooling, extract was filtered using Whatman filter paper No. 1, then the extract was evaporated under vacuum. The dried extract was dissolved in 1 mL distilled deionized water. The precolumn derivatization with OPA reagent was carried out according to Wang et al. [129] method as follows: 70 μL extract was derivatized with 10 μL of OPA reagent (*O*-phthalaldehyde and 3-mercaptopropionic acid) at 25 °C and pH of 9.5 for 2 min. This mixture was immediately separated by HPLC.

HPLC analysis was carried out according to Henderson and Brooks [130]. The method was used an Agilent 1260 series. The separation was carried out using Eclipse Plus C18 column (4.6 × 250 mm i.d., 5 μm). The mobile phase consisted of solvent A (buffer sodium phosphate buffer pH 7.8) and solvent B (acetonitrile: methanol: water 45:45:10 *v*/*v*) at a flow rate 1.5 mL/min. The mobile phase was programmed consecutively in a linear gradient as shown in the following Table 6:

The flow rate was 0.8 mL/min, injection volume was 10 μL, and the column temperature was maintained at 40 °C. The analysis was monitored by DAD at 338 nm (bandwidth 10 nm). The fluorescence detector was adjusted as the following: from 0 to 25 min at 340/450 nm (Excitation/Emission) and from 25 to 40 min at 266/305 nm (Excitation/Emission).

### 4.5. Phenolic Compounds Separation by HPLC

To measure total phenolic compounds via HPLC, 0.2 g of fresh tissue was soaked in 1 mL HPLC-grade methanol then centrifuged, and the supernatant was used in HPLC analysis. The separation with HPLC was carried out according to Gökbulut [131], using an Agilent 1260 series. The separation was carried out using Eclipse C18 column (4.6 × 250 mm i.d., 5 μm). The mobile phase consisted of water (A) and 0.05% trifluoroacetic acid (Sigma-Aldrich, Merck, Germany) in acetonitrile (B) (HPLC-grade 99.9%) at a flow rate of 0.9 mL/min. The mobile phase was programmed consecutively in a linear gradient as follows: 0 min (82% A); 0–5 min (80% A); 5–8 min (60% A); 8–12 min (60% A); 12–15 min (82% A); 15–16 min (82% A) and 16–20 (82% A). The multi-wavelength detector was monitored at 280 nm. The injection volume was 5 μL for each of the sample solutions. The column temperature was maintained at 40 °C.

### 4.6. Alkaloids (Vincamine, Catharanthine, Vincracine (Vincristine) and Vinblastine) Analysis by HPLC

The detection of alkaloids (Vincamine, Catharanthine, Vincracine (Vincristine) and vinblastine) were performed using HPLC according to Liu et al. [132]. About 10 g sample transferred into 10 mL centrifuge tube and then soaked with 10 extracting solution (2% formic acid: methanol (50:50 *v*/*v*) for 2 h. The mixture was sonicated for 30 min and centrifuged for 10 min at 4000 rpm. The supernatant was transferred into another tube filtered with 0.2 um PTFE syringe filter and injected in HPLC. Agilent 1260 infinity HPLC Series (Agilent, Santa Clara, CA 95051, USA), equipped with Quaternary pump, the column used: zorbax Eclipse plus C18 150 × 4.6 mm was operated at 30 °C. The separation was achieved using a ternary linear isocratic elution with (A) HPLC-grade water 0.2% formic (*v*/*v*), (B) methanol as mobile phase at a flow rate 1 mL/min. A VWD detector was used and the wavelength 254 nm was selected for the analysis.

### 4.7. Statistical Analysis

SAS [133] software was used to do a one-way ANOVA procedure. Means ± standard deviations (SD) from three replicates were calculated, and the Tukey’s Studentized Range (HSD) Test (*p* < 0.05) was performed to evaluate significant differences between means.

## 5. Conclusions

Foliar application with ĸ-carrageenan treatments enhances plant height, leaf area, branches, leaves and flowers numbers, which leads to growth improvement. Additionally, carrageenan treatments have positive effects on important phytochemical consitituents such as phenolic compounds and flavonoids, which have high antioxidant and anticancer activity. However, they especially elevate total alkaloids content. Vincristine, which is considered the most common alkaloids of vinca, has a broad uses in pediatric cancer.

## Figures and Tables

**Table 1:** Effect of carrageenan spraying on phytochemical constituents of *Catharanthus roseus* leaves expressed as equivalents of standard for each assay.

Carrageenan Treatments	TPC	TFC	TAC	FAA
	μg Gallic/g FW	μg Quercetin/g FW	μg Atropine/g DW	mg Lysine/g FW
0	2476.5 ^d^ ± 5.711	271.7 ^d^ ± 1.815	1359 ^d^ ± 2.645	132.05 ^c^ ± 4.173
400 ppm	3066.4 ^c^ ± 1.742	466.1 ^c^ ± 2.775	2535 ^c^ ± 0.577	195.57 ^a^ ± 3.115
600 ppm	3425.6 ^b^ ± 3.009	663.6 ^b^ ± 3.671	3064 ^b^ ± 4.509	138.07 ^bc^ ± 3.684
800 ppm	3948.6 ^a^ ± 3.492	754.6 ^a^ ± 3.329	3518 ^a^ ± 0.046	145.49 ^bc^ ± 1.061

TPC = total phenolic content, TFC = total flavonoid content, TAC = total alkaloid content, FAA = free amino acids. Different letters indicate significant differences according to Duncan’s multiple range tests (*p* < 0.05).

**Table 2:** Effect of ĸ-carrageenan on content of elements in *Catharanthus roseus*.

Elements Concentration	Carrageenan Treatments			
	0 ppm	400 ppm	600 ppm	800 ppm
**N** (mg/Kg DW)	15.58 ^b^ ± 0.196	13.36 ^d^ ± 0.213	14.66 ^c^ ± 0.125	16.42 ^a^ ± 0.1457
**P** (g/100 g DW)	0.063 ^a^ ± 0.000	0.066 ^a^ ± 0.001	0.060 ^a^ ± 0.000	0.067 ^a^ ± 0.0002
**K** (g/100 g DW)	0.29 ^d^ ± 0.004	0.33 ^b^ ± 0.009	1.38 ^a^ ± 0.010	1.23 ^c^ ± 0.0059
**Ca** (g/100 g DW)	0.25 ^c^ ± 0.003	1.54 ^b^ ± 0.002	1.65 ^a^ ± 0.042	1.63 ^a^ ± 0.0295
**Mn** (mg/Kg DW)	76.98 ^c^ ± 0.027	46.14 ^d^ ± 0.380	154.56 ^a^ ± 0.169	128.79 ^b^ ± 0.7794
**Cu** (mg/Kg DW)	7.48 ^c^ ± 0.397	78.79 ^a^ ± 0.417	13.92 ^b^± 0.4769	12.50 ^b^ ± 0.4392
**Zn** (mg/Kg DW)	15.00 ^c^ ± 0.987	54.22 ^a^ ± 0.129	43.80 ^b^ ± 0.2810	43.96 ^b^ ± 0.8215
**Se** (mg/Kg DW)	32.43 ^d^ ± 0.282	117.17 ^a^ ± 0.075	92.46 ^b^ ± 1.0016	85.40 ^c^ ± 0.0139

Different letters indicate significant differences according to Duncan’s multiple range tests (*p* < 0.05).

**Table 3:** HPLC analysis of free amino acids in *Catharanthus roseus* leaves treated with ĸ-carrageenan.

	Amino Acids (mg/g FW)	Rt (min)	Carrageenan Treatments			
			0 ppm	400 ppm	600 ppm	800 ppm
1	ASP	2.973	1.55 ^a^ ± 0.011	1.32 ^b^ ± 0.004	0.91 ^c^ ± 0.003	0.78 ^d^ ± 0.004
2	GLU	4.721	4.01 ^a^ ± 0.028	1.84 ^d^ ± 0.010	2.18 ^c^ ± 0.027	2.78 ^b^ ± 0.021
3	Serine	8.391	5.41 ^a^ ± 0.039	5.28 ^b^ ± 0.034	5.06 ^c^ ± 0.041	5.27 ^b^ ± 0.050
4	Histidine	10.248	0.23 ^c^ ± 0.001	0.25 ^b^ ± 0.002	0.18 ^d^ ± 0.001	0.26 ^a^ ± 0.001
5	Glycine	10.566	1.48 ^c^ ± 0.009	1.60 ^bc^ ± 0.008	1.70 ^ab^ ± 0.007	1.85 ^a^ ± 0.005
6	Threonine	10.85	4.03 ^d^ ± 0.054	4.58 ^b^ ± 0.050	4.45 ^c^ ± 0.049	6.82 ^a^ ± 0.060
7	Arginine	12.621	9.21 ^a^ ± 0.104	3.08 ^c^ ± 0.047	0.614 ^d^ ± 0.003	4.32 ^b^ ± 0.052
8	Alanine	13.122	6.85 ^a^ ± 0.056	4.14 ^b^ ± 0.053	2.05 ^c^ ± 0.017	1.23 ^d^ ± 0.009
9	Tyrosine	15.52	3.14 ^a^ ± 0.066	2.48 ^d^ ± 0.023	3.11 ^b^ ± 0.031	2.73 ^c^ ± 0.048
10	Cysteine	17.349	----	----	---	----
11	Valine	18.783	3.52 ^d^ ± 0.017	3.73 ^c^ ± 0.021	4.17 ^b^ ± 0.041	4.93 ^a^ ± 0.050
12	Methionine	19.191	4.31 ^c^ ± 0.029	4.34 ^c^ ± 0.051	4.58 ^b^ ± 0.039	5.00 ^a^ ± 0.051
13	Tryptophan	20.796	1.06 ^d^ ± 0.008	1.11 ^c^ ± 0.009	1.43 ^b^ ± 0.011	2.44 ^a^ ± 0.014
14	Phenylalanine	21.478	3.75 ^d^ ± 0.022	4.28 ^a^ ± 0.044	3.84 ^c^ ± 0.033	4.07 ^b^ ± 0.035
15	IsoLeucine	21.817	2.51 ^d^ ± 0.019	3.36 ^a^ ± 0.023	3.06 ^c^ ± 0.043	3.16 ^b^ ± 0.031
16	Leucine	23.018	5.28 ^d^ ± 0.031	6.32 ^a^ ± 0.048	5.57 ^c^ ± 0.047	6.23 ^b^ ± 0.051
17	Lysine	24.296	1.42 ^a^ ± 0.006	0.25 ^d^ ± 0.001	0.82 ^b^ ± 0.002	0.54 ^c^ ± 0.001
18	Proline	29.288	56.45 ^d^ ± 1.025	59.82 ^c^ ± 1.030	62.98 ^b^ ± 1.007	65.28 ^a^ ± 1.072

Different letters indicate significant differences according to Duncan’s multiple range tests (*p* < 0.05).

**Table 4:** HPLC analysis of phenolic compounds in *Catharanthus roseus* leaves treated with ĸ-carrageenan.

	Phenolic Compounds (mg/g FW)	R_t_ min	0 ppm	400 ppm	600 ppm	800 ppm
1	Gallic acid	3.526	0.93 ^a^ ± 0.004	0.71 ^b^ ± 0.003	0.72 ^b^ ± 0.003	0.72 ^b^ ± 0.004
2	Chlorogenic acid	4.311	11.18 ^d^ ± 0.521	15.47 ^c^ ± 0.601	17.92 ^b^ ± 0.624	18.97 ^a^ ± 0.671
3	catechin	4.622	---	---	---	---
4	Methyl gallate	5.401	3.58 ^b^ ± 0.021	5.16 ^a^ ± 0.033	2.65 ^c^ ± 0.012	2.19 ^d^ ± 0.012
5	Caffeic acid	5.8820	0.64 ^a^ ± 0.003	0.55 ^b^ ± 0.004	0.48 ^c^ ± 0.002	0.40 ^d^ ± 0.003
6	Syringic acid	6.611	3.13 ^d^ ± 0.014	4.98 ^a^ ± 0.021	4.11 ^c^ ± 0.024	4.26 ^b^ ± 0.026
7	pyrocatechol	6.791	---	---	---	---
8	Rutin	7.839	0.33 ^b^ ± 0.001	1.11 ^a^ ± 0.009	0.19 ^c^ ± 0.001	0.20 ^c^ ± 0.001
9	Ellagic acid	9.036	---	0.89 ^a^ ± 0.002	0.30 ^b^ ± 0.001	---
10	Coumaric acid	9.074	0.15 ^c^ ± 0.001	0.15 ^c^ ± 0.001	0.18 ^b^ ± 0.002	0.22 ^a^ ± 0.002
11	Vanillin	9.774	0.10 ^b^ ± 0.001	0.22 ^a^ ± 0.002	0.12 ^b^ ± 0.001	0.23 ^a^ ± 0.002
12	Ferulic acid	10.292	0.23 ^d^ ± 0.001	0.30 ^c^ ± 0.002	0.49 ^b^ ± 0.002	0.74 ^a^ ± 0.004
13	Naringenin	10.587	0.11 ^c^ ± 0.001	0.14 ^c^ ± 0.001	0.27 ^b^ ± 0.001	0.30 ^a^ ± 0.001
14	Daidzein	12.12	0.07 ^d^ ± 0.001	1.38 ^c^ ± 0.025	1.59 ^b^ ± 0.027	1.88 ^a^ ± 0.030
15	Querectin	12.545	1.10 ^d^ ± 0.027	1.97 ^c^ ± 0.030	3.10 ^b^ ± 0.040	3.73 ^a^ ± 0.043
16	Cinnamic acid	14.014	0.02 ^c^ ± 0.001	0.06 ^c^ ± 0.001	0.16 ^b^ ± 0.001	0.24 ^a^ ± 0.001
17	Apigenin	14.514	0.03 ^a^ ± 0.001	---	---	---
18	kaempferol	15.024	---	---	---	---
19	hesperetin	15.606	---	---	---	---

Different letters indicate significant differences according to Duncan’s multiple range tests (*p* < 0.05).

**Table 5:** Effect of ĸ-carrageenan treatments on alkaloids contents of Catharanthus roseus leaves identified by HPLC.

Alkaloid Compounds μg/g	Treatments			
	0	400 ppm	600 ppm	800 ppm
vincamine	24.911	12.043	16.227	18.167
catharanthine	9.878	1.559	1.662	10.157
Vincracine (Vincristine)	-	6.574	9.129	13.522
vinblastine	-	-	-	-
Total	34.789	20.176	27.018	41.846

**Table 6:** Mobile phase program at HPLC analysis of amino acids.

Time (Min)	A%	B%
0	98	2
0.84	98	2
33.40	43	57
33.50	0	100
39.30	0	100
39.40	98	2
40.0	98	2

## Data Availability

All data are available within the manuscript.

## Supplementary Materials

The following supporting information can be downloaded at: https://www.mdpi.com/article/10.3390/molecules28083642/s1. HPLC—Analysis chromatograms of *Catharanthus roseus* Leaf alkaloids.
