# Supplementary material for: Potentiating Biosynthesis of Alkaloids and Polyphenolic Substances in Catharanthus roseus Plant Using ĸ-Carrageenan
Source: Molecules. 2023 Apr 21;28(8):3642. doi: 10.3390/molecules28083642 (PMC10143362; doi:10.3390/molecules28083642)
Supplement: Supplementary file 1 [file molecules-28-03642-s001.zip › molecules-2286661-supplementary.pdf]

Short Report (ESTD)

Agilent Technologies

Data file: D:\CHEM32\1\DATA\ALKALOIDS\FSQC0147-2023 1405 2023-04-1110-52-29.D
Sample name: FSQC0147-2023
Description:

Instrument: FSQC-HPLC1260
Injection date: 4/11/2023 10:52:29 AM
Acq. method: VINBLASTINE_LC.M
Analysis method: VINBLASTINECAL _LC.M **Last changed:** 4/11/2023 1:17:10 PM
Sample type: Sample **ID:**
Injection volume: No injector **Sample amount:** 10.000 **Dilution:** 2
Multiplier: 1

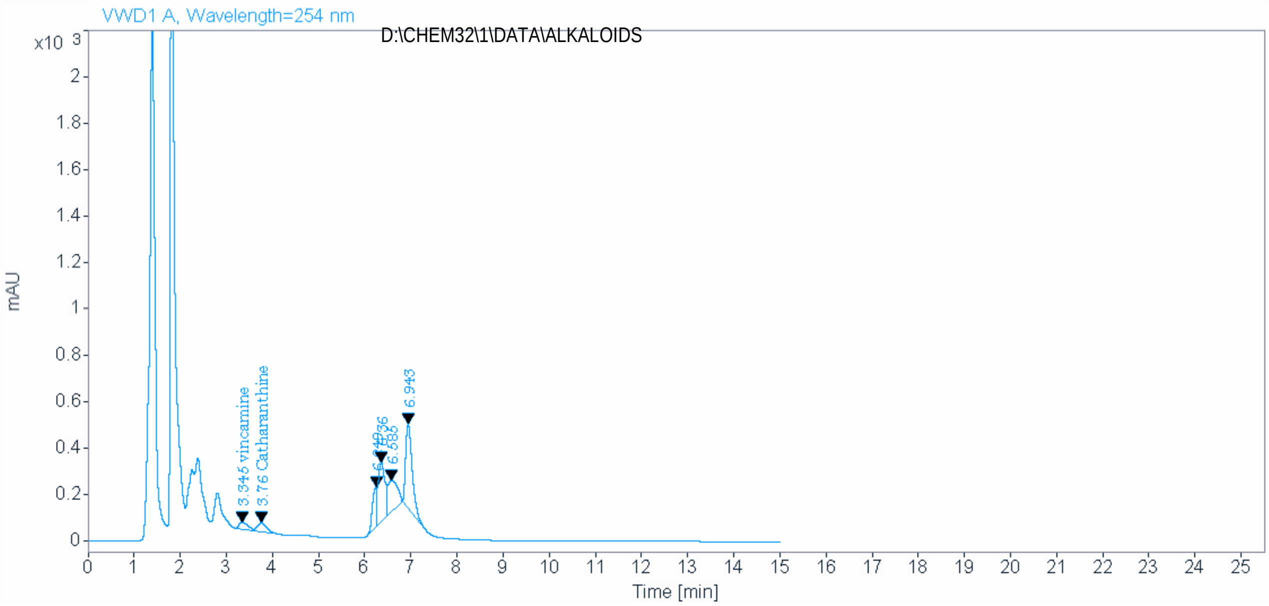

Signal: VWD1 A, Wavelength=254 nm

Name	Compound_ExpectedRetTime	RT [min]	Area	Amount [ug/g]
vincamine	3.280	3.35	425.6965	24.911
Catharanthine	3.900	3.76	557.7837	9.878
			Number of	2.0
			Max	24.91
			Min	9.88

Compound Name	Expected RT
Vinblastin	13.99
Vinracine	5.89

Short Report (ESTD)

Agilent Technologies

Data file: D:\CHEM32\1\DATA\ALKALOIDS\FSQC0148-2023 1402 2023-04-1013-03-07.D
Sample name: FSQC0148-2023
Description:

**Instrument:**	FSQC-HPLC1260				
**Injection date:**	4/10/2023 1:03:07 PM				
**Acq. method:**	VINBLASTINE_LC.M				
**Analysis method:**	VINBLASTINECAL _LC.M	**Last changed:**	4/11/2023 1:15:36 PM		
**Sample type:**	Sample	**ID:**			
**Injection volume:**	No injector	**Sample amount:**	10.000	**Dilution:**	2
**Multiplier:**	1				

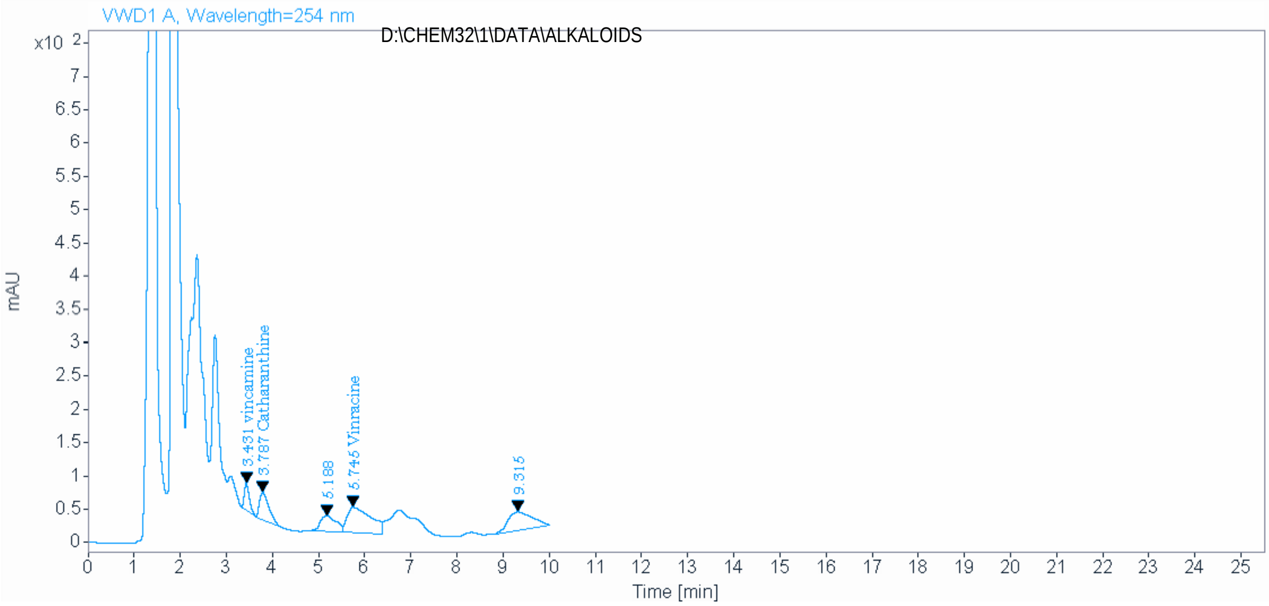

Signal: VWD1 A, Wavelength=254 nm

Name	Compound_ExpectedRetTime	RT [min]	Area	Amount [ug/g]
vincamine	3.280	3.43	310.4533	18.167
Catharanthine	3.900	3.79	575.6954	10.157
Vinracine	5.890	5.75	1373.0406	13.522
			Number of	3.0
			Max	18.17
			Min	10.16

Compound Name	Expected RT
Vinblastin	13.99

Short Report (ESTD)

Agilent Technologies

**Data file:**	D:\CHEM32\1\DATA\ALKALOIDS\FSQC0149-2023 1407 2023-04-1111-28-28.D				
**Sample name:**	FSQC0149-2023				
**Description:**					
**Instrument:**	FSQC-HPLC1260				
**Injection date:**	4/11/2023 11:28:28 AM				
**Acq. method:**	VINBLASTINE_LC.M				
**Analysis method:**	VINBLASTINECAL_LC.M	**Last changed:**	4/11/2023 1:07:04 PM		
**Sample type:**	Sample	**ID:**			
**Injection volume:**	No injector	**Sample amount:**	10.000	**Dilution:**	2
**Multiplier:**	1				

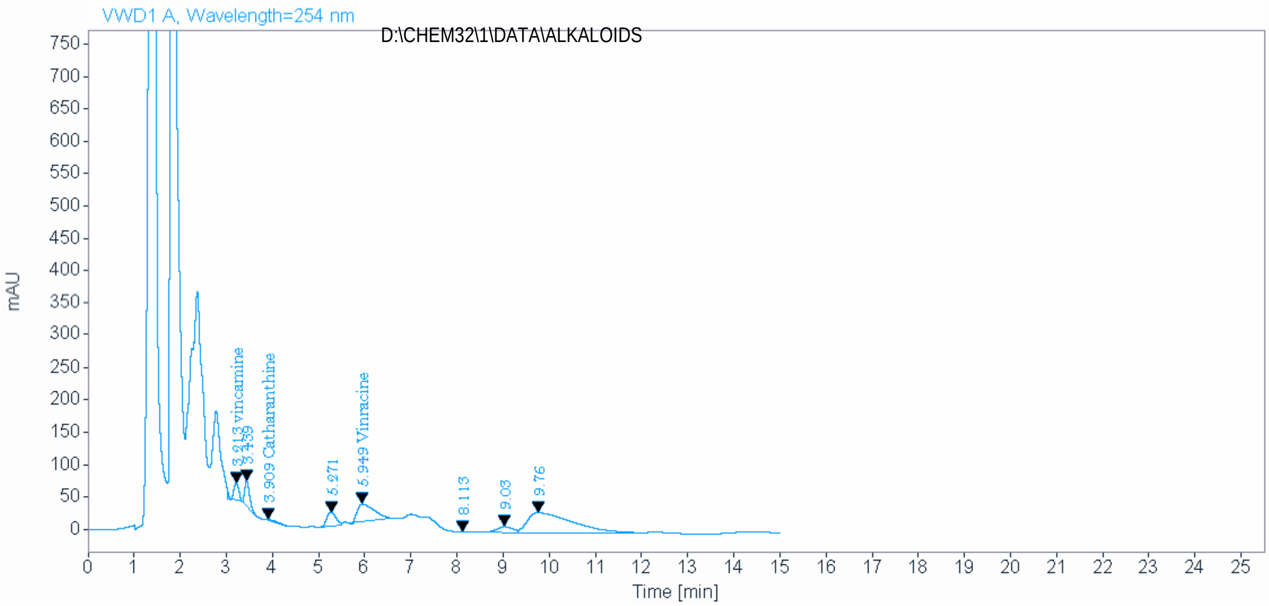

Signal: VWD1 A, Wavelength=254 nm

Name	Compound_ExpectedRetTime	RT [min]	Area	Amount [ug/g]
vincamine	3.280	3.21	205.7971	12.043
Catharanthine	3.900	3.91	25.4016	1.559
Vinracine	5.890	5.95	659.5424	6.574
			Number of	3.0
			Max	12.04
			Min	1.56

Compound Name	Expected RT
Vinblastin	13.99

Short Report (ESTD)

Agilent Technologies

**Data file:**	D:\CHEM32\1\DATA\ALKALOIDS\FSQC0150-2023 1406 2023-04-1111-10-46.D
**Sample name:**	FSQC0150-2023
**Description:**	

**Instrument:**	FSQC-HPLC1260				
**Injection date:**	4/11/2023 11:10:46 AM				
**Acq. method:**	VINBLASTINE_LC.M				
**Analysis method:**	VINBLASTINECAL _LC.M	**Last changed:**	4/11/2023 1:21:55 PM		
**Sample type:**	Sample	**ID:**			
**Injection volume:**	No injector	**Sample amount:**	10.000	**Dilution:**	2
**Multiplier:**	1				

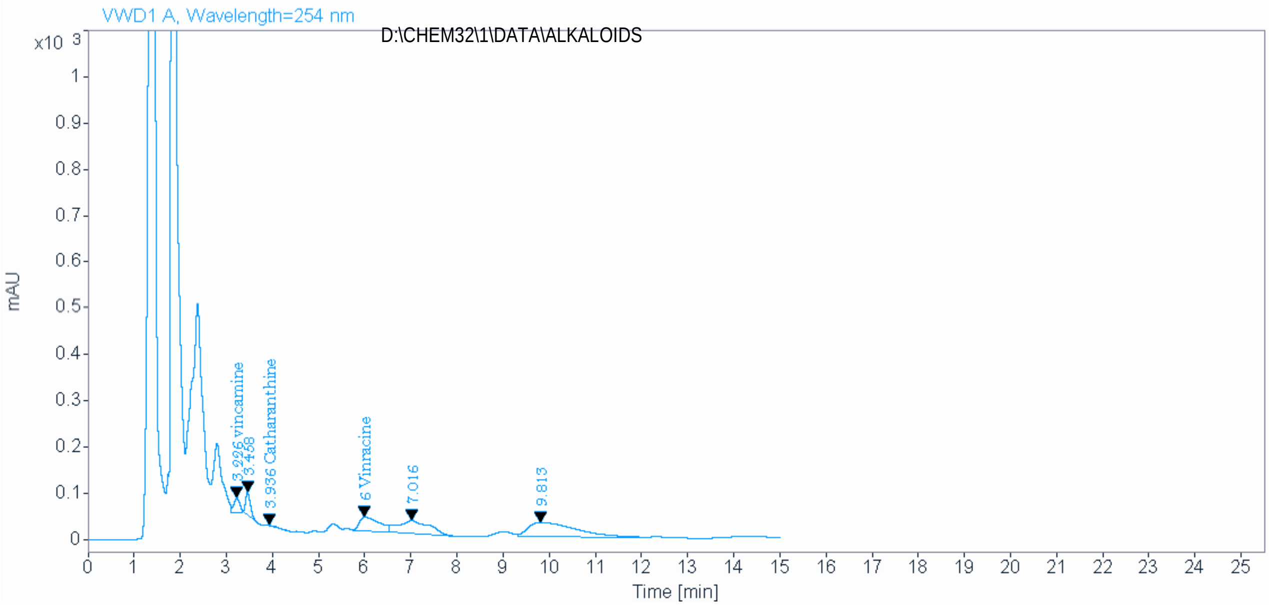

Signal: VWD1 A, Wavelength=254 nm

Name	Compound_ExpectedRetTime	RT [min]	Area	Amount [ug/g]
vincamine	3.280	3.23	277.2920	16.227
Catharanthine	3.900	3.94	32.0091	1.662
Vinracine	5.890	6.00	921.9019	9.129
			Number of	3.0
			Max	16.23
			Min	1.66

Compound Name	Expected RT
Vinblastin	13.99
